# Supplementary material for: Snapshot multispectral imaging using a diffractive optical network
Source: Light Sci Appl. 2023 Apr 6;12:86. doi: 10.1038/s41377-023-01135-0 (PMC10079962; doi:10.1038/s41377-023-01135-0)
Supplement: Supplementary file 1 — Supplementary Information [file 41377_2023_1135_MOESM1_ESM.pdf]

Supplementary Information for

Snapshot Multispectral Imaging Using a Diffractive Optical Network

Deniz Mengu[1,2,3], Anika Tabassum[1,2,3], Mona Jarrahi[1,2,3], Aydogan Ozcan[1,2,3,*]

[1] Electrical and Computer Engineering Department, University of California, Los Angeles, CA, 90095, USA

[2] Bioengineering Department, University of California, Los Angeles, CA, 90095, USA

[3] California NanoSystems Institute, University of California, Los Angeles, CA, 90095, USA

[§] Equal contribution

* Corresponding author: ozcan@ucla.edu

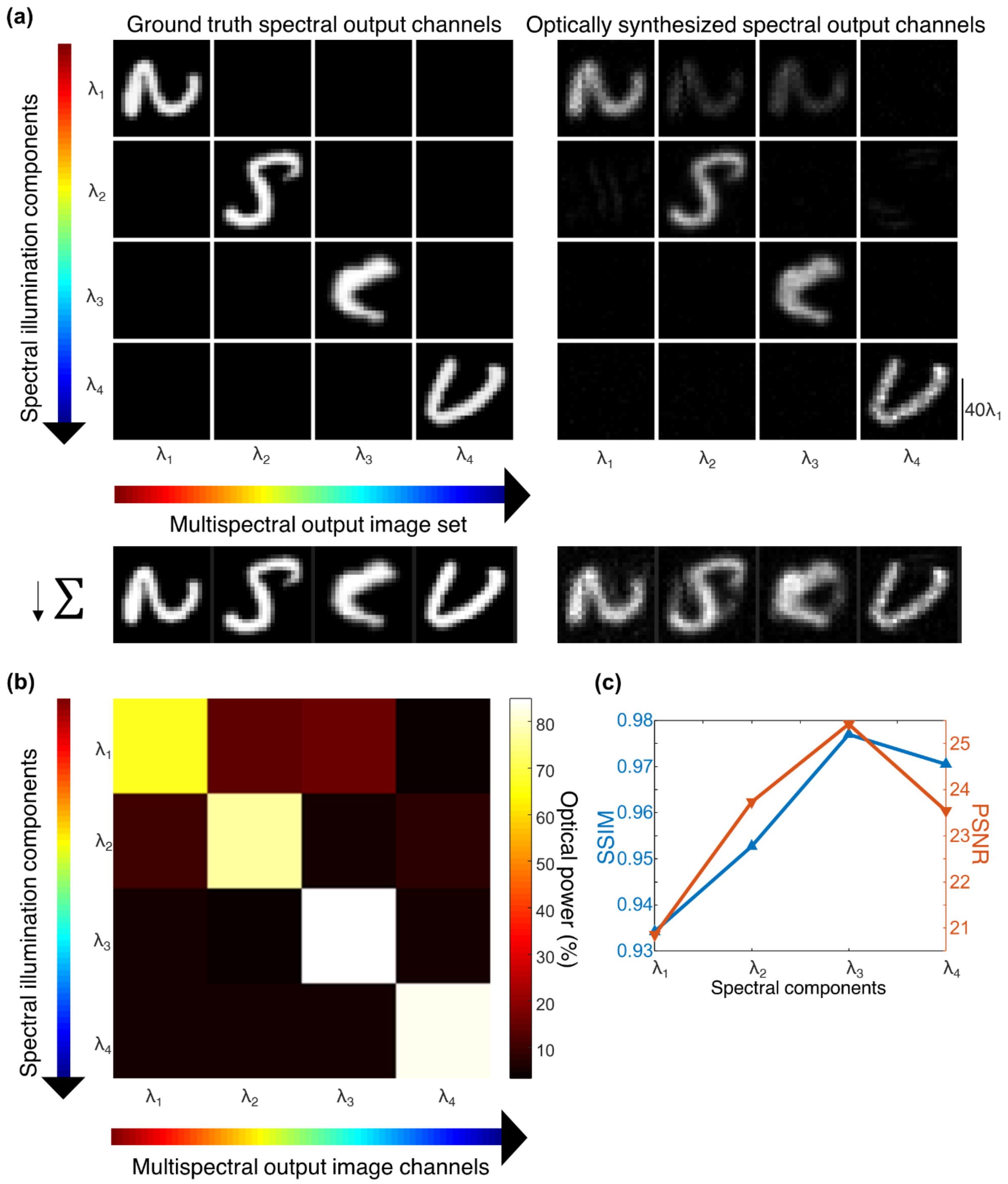

Figure S1. Performance of a diffractive multispectral imager with $N_B = 4$ spectral bands. a-c Same as Figure 2 (main text), except for $N_B = 4$.
